# Not Just Rocked, but Delighted: Rhythmic Passive Movement Induces Positive States in Chicks

**DOI:** 10.1111/nyas.70321

**Published:** 2026-06-16

**Authors:** Cinzia Chiandetti, Andrea Dissegna, Lorenzo Scalera, Paolo Gallina

**Affiliations:** ^1^ Department of Life Sciences University of Trieste Trieste Italy; ^2^ Polytechnic Department of Engineering and Architecture University of Udine Udine Italy; ^3^ Department of Engineering and Architecture University of Trieste Trieste Italy

**Keywords:** affective state, arousal, carrying, *Gallus gallus domesticus*, locomotor play, rocking, vocalizations

## Abstract

Rhythm is a fundamental organizing principle in biological systems, shaping physiological regulation, affect, and social interaction across species. In caregiving, rhythmic passive movement, such as carrying and rocking, has long been recognized as a soothing strategy in mammals, reducing crying, heart rate, and arousal while promoting sleep. Anecdotal and behavioral evidence in humans also suggest that some forms of rhythmic motion, such as sliding or tossing infants, can elicit overt enjoyment. Yet, no study has directly investigated the effects of passive rhythmic movement beyond the mammalian lineage or in species lacking maternal carrying, nor assessed its potential to evoke pleasurable states. Here, we examined the effects of rhythmic passive movement in domestic chicks (*Gallus gallus*), a precocial bird that walks immediately after hatching and is never carried by the mother. Chicks were exposed to rocking (horizontal, vertical) and carrying‐like motion at different frequencies. Slow rhythmic movements reduced contact calls, replicating the soothing effects known in mammals, while faster movements increased brood calls, indicating a transition from distress reduction to positive arousal. These results demonstrate that rhythmic passive motion modulates affective states in birds, revealing both calming and pleasurable dimensions and suggesting deeply conserved mechanisms for rhythm‐based affect regulation across vertebrates.

## Graphical abstract

Rhythmic passive movement modulates affective states in domestic chicks, a species without maternal carrying. Slow rocking‐like and carrying‐like movements reduce contact vocalizations, reproducing mammalian soothing effects, while fast horizontal rocking‐like and fast carrying‐like movements increase pleasure‐related calls. These findings demonstrate that rhythmic motion can induce both calming and pleasurable states, revealing conserved vertebrate mechanisms linking rhythm, movement, and affect regulation.

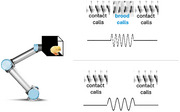

## 1 Introduction

Rhythm is a fundamental organizing principle in biological systems: it underlies physiological regulation, perception, and social interaction, and extends to domains such as communication, music, and collective behavior [1, 2]. Rhythmic passive motion has long been recognized as a powerful soothing cue in caregiving [3, 4]. Mammals commonly employ regular carrying and rocking to alleviate distress, translating mechanosensory stimulation into regulation of affective states [5]. Documented effects include reductions in crying, heart rate, and arousal, as well as facilitation of sleep, underscoring rhythm's role in coordinating internal states and social behavior across species [6].

But which features of rhythmic motion are critical? Rocking and carrying can vary in frequency (fast and slow), direction (horizontal and vertical), and pattern (continuous and intermittent), and may or may not be accompanied by maternal contact. Early studies showed that higher rocking frequencies produced stronger calming effects [7, 8]. Vertical rocking was reported to be particularly effective [9], though its effects faded after motion ceased, unlike horizontal rocking, which persisted into the post‐rocking phase [10]. Continuous rocking also enhanced infant sleep more than intermittent rocking [11]. Previous literature on infant carrying reported conflicting results, largely because they relied on parental diaries over extended periods and did not distinguish between active carrying and static holding [3, 12]. However, Esposito and collaborators [13] showed that in human infants under 6 months of age, being carried by a walking mother, thus also with direct contact, induces immediate cessation of voluntary movement and crying, together with a rapid decrease in heart rate, compared with static holding. Remarkably similar responses are seen in mouse pups, where carrying triggers immobility, suppression of ultrasonic vocalizations, and cardiac slowing. It has also been shown that somatosensory and proprioceptive inputs are necessary for their induction, with parasympathetic and cerebellar pathways mediating the motor and cardiac components. The loss of this response in the offspring hinders maternal rescue, thus highlighting the adaptive significance of mother−infant coordination linked to carrying.

Despite these advances in the understanding of basic principles governing rocking and carrying, no research has directly addressed the question of when and how these movements shift from merely soothing to being a pleasurable condition. Indeed, everyday experience suggests that rhythmic passive motion is not limited to calming contexts: parents often toss or gently throw babies in playful ways, and children derive evident enjoyment from rides such as roller coasters, swings, and slides. Such affective states go beyond the dimension of distress attenuation, entering that of positive arousal and enjoyment [14−16]. Repeated and apparently purposeless motion in nonhuman animals is viral on social media: for example, sliding behavior in elephants, pandas, and several bird species strongly resembles human play on slides and swings, suggesting that locomotor play is associated with pleasure [17, 18]. Thus, we wondered whether these instances highlight how rhythmic or dynamic motion, both self‐generated and externally imposed, may recruit mechanisms of positive affect that overlap those elicited by rocking and carrying.

Addressing these issues would help elucidating how rhythmic motion shapes arousal regulation and pleasurable state across species; therefore, we investigated the effects of rocking and carrying‐like movements on affective responses in domestic chicks (*Gallus gallus*) using vocalizations as a well‐established, noninvasive indicator of affective states [19−21]. This model offers a unique comparative opportunity: as a precocial bird species, chicks are capable of independent locomotion at hatching yet are not naturally exposed to maternal carrying, allowing us to dissociate the effects of rhythmic passive motion from maternal caregiving. Our approach was also motivated by repeated empirical observations during routine handling, whereby chicks frequently showed a rapid reduction in agitation and vocalizations when transported in opaque boxes by the walking operator from the rearing to the experimental room, suggesting that passive movement itself may influence affective state even in the absence of social contact. Indeed, we focused on walking, as inherently rhythmic and regular, generating predictable oscillatory vestibular and proprioceptive inputs [22]. Demonstrating modulation of affect in the domestic chick would support the view that sensitivity to rhythmic passive motion reflects a conserved regulatory mechanism extending beyond the mammalian lineage.

## 2 Experiment 1

### 2.1 Materials and Methods

#### 2.1.1 Animals

We observed 135 two‐day‐old domestic chickens (*Gallus gallus domesticus*, Ross 308 broiler strain, Aviagen); 68 chicks were female, and 67 were male. Chicks were hatched from eggs sourced from a commercial poultry hatchery (Agricola Berica scrl, Montegalda, Vicenza, Italy) and incubated under controlled conditions of 37.7°C and 50% relative humidity within FIEM incubators. After hatching, the chicks were individually reared in a dedicated room with a 12:12 light/dark cycle and a constant temperature of 31°C within rectangular cages (22, 30, 40 cm, width, height, depth) with a cylindric imprinting object until the day of the experiment serving as social enrichment; chick crumbles and water were available *ad libitum*.

#### 2.1.2 Procedure

**FIGURE 1:**
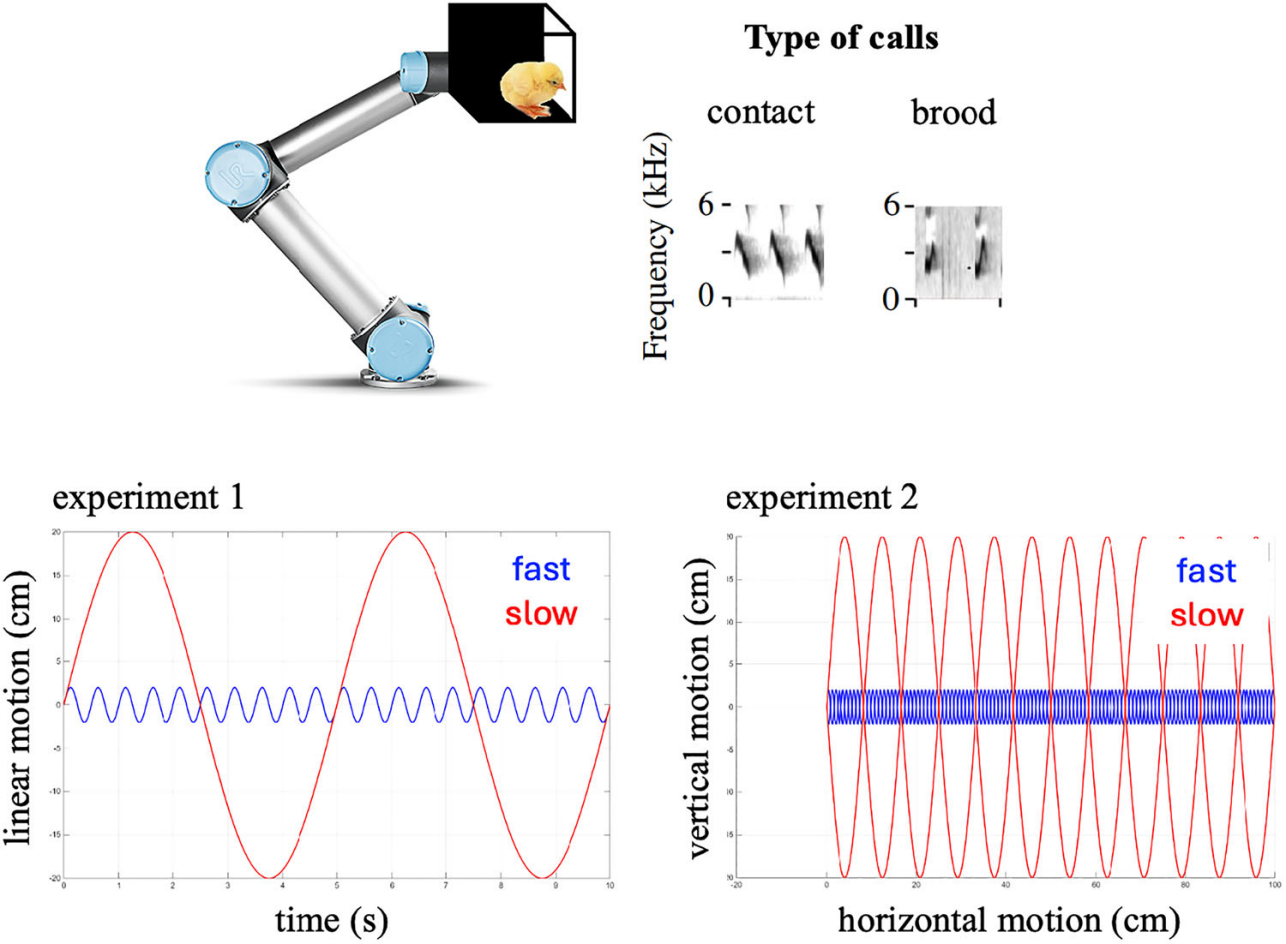
Schematic layout of the apparatus with the robotic arm, and the holding box for the chick with types of calls recorded.

The experimental apparatus consisted of an opaque square box (30 cm width, height, depth) mounted at the end of a robotic arm (UR 10/CB3, Universal Robots, see Figure 1). The arm offered a 1300‐mm working radius in all directions and ensured a positioning repeatability of ±0.1 mm, allowing for high movement precision along both the vertical and horizontal axes, as well as in their combined motion. The arm position was controlled via a custom‐made MATLAB program. Rocking was generated by operating the robotic arm along either the horizontal or vertical axis. Two conditions were defined based on movement frequency and displacement: (1) a fast‐rocking condition, characterized by a 2 Hz movement over a 2 cm displacement, and (2) a slow‐rocking condition, characterized by a 0.2 Hz movement over a 20 cm displacement. The movements of the robot are defined by horizontal linearity combined with vertical sinusoidal patterns. In this case, the maximum vertical acceleration is provided by the formula $a_{max}=A\;{\left(2\pi f\right)}^{2}$ with *a_max_* ​ indicating the peak acceleration reached during the oscillatory motion, *A* representing the maximum distance from the central position reached during the oscillation, and 2*πf* expressing the angular frequency of the oscillation. The two conditions of fast rocking and slow rocking, therefore, produce accelerations of 316 and 32 cm/s^2^, respectively. The selection of the acceleration range used in the current experiments was grounded in prior evidence demonstrating that rocking efficacy scales with the maximum linear acceleration. Previous human studies reported behavioral effects at accelerations between approximately 15 and 26 cm/s^2^, while more recent mouse data [22] indicated that sleep modulation emerges at ∼79 cm/s^2^ and increases with higher accelerations, with 32 cm/s^2^ representing a threshold at which no significant effect was observed. Importantly, mouse vestibular afferents are estimated to be three to four times less sensitive than those of primates, suggesting that effective accelerations in mice correspond to proportionally lower values in humans. On this basis, we selected 32 cm/s^2^ as a lower‐bound condition approximating the subthreshold or minimally effective range, and 316 cm/s^2^ as a suprathreshold condition well above previously reported effective values (79–178 cm/s^2^), ensuring robust stimulation. This wide dynamic range allows us to probe both threshold‐level and strongly activating regimes of linear acceleration, thereby testing the predicted dependence of the rocking effect on peak acceleration magnitude while accounting for known interspecies sensitivity differences.

Individual chicks were placed in the robotic arm's enclosure and allowed to acclimate to this new environment for 1 min before the experiment began. The experiment comprised three phases, each lasting 1 min: (1) a pre‐rocking still phase, with the arm stationary in the starting position; (2) a rocking phase, in which the arm moved according to one of the defined rocking conditions; and (3) a post‐rocking still phase identical to the pre‐rocking one.

Chicks were randomly assigned to one of the four rocking conditions (fast or slow rocking, applied along either the vertical or horizontal axis; *n* = 27 per condition). An additional control group (*n* = 27) was tested with the robotic arm kept stationary throughout the experiment.

We recorded chicks’ vocalizations during three phases via an AKG C1000S Microphone (Wien, Austria) fixed on the external wall of the robotic arm's box. The recorded audio was digitally converted by a Focusrite Scarlett 2i4 audio card (High Wycombe, UK) connected to a computer (sampling rate: 44.1 kHz; amplitude resolution: 16 bits). Data are available in the Supplementary Materials.

We analyzed the number of contact calls and brood calls emitted by chicks. These two call types represent major stereotyped vocalizations crucial for communication between a chick and its mother, its imprinting object, or another chick, and are considered a robust noninvasive indicator of affective states in chicks [19, 21]. Contact calls are characterized by a descending fundamental frequency (Figure 1), and are emitted by chicks when they experience discomfort, especially when they are separated from the hen, their imprinting object, or their brood, and are exposed to threats or aversive stimulation [19−21, 23, 24]. By contrast, brood calls contain both ascending and descending frequencies (Figure 1), occur in affiliative or nondistress contexts in which arousal levels are low to moderate, such as during gentle social interactions with conspecifics, mild handling, or exploratory situations [20, 24, 25]. These vocalizations are also observed at pre‐hatching stages when the mother hen approaches the eggs: in such situations, embryos may become silent or begin emitting brood‐type vocalizations [26].

#### 2.1.3 Data Analysis

Audio recordings were analyzed in Praat version 6.0.1. The spectrogram of each recorded audio was visually inspected to detect contact and brood calls by two independent raters. Interobserver agreement for the classification of chick vocalizations into contact calls and pleasure calls was assessed using Cohen's *κ* based on 540 vocalizations. We performed separate mixed factors ANOVAs to test for differences in the number of contact and brood calls across phases (pre‐rocking, rocking, and post‐rocking) and rocking conditions (fast vertical, fast horizontal, slow vertical, slow horizontal, and control). We reported Greenhouse−Geisser corrected results whenever Mauchly's test returned a violation of the sphericity assumption. Post‐hoc comparisons were conducted using Tukey's *t‐*tests. As estimates of the effect size, we provided partial eta squared ($\eta_{p}^{2}$) for the interactions and main effects of the ANOVA *F*‐tests. Data were analyzed using R, version 4.5.1 [27] and the packages *dplyr* [28], *tidyr* [29], *ggplot2* [30], *afex* [31], and *emmeans* [32].

### 2.2 Results and Discussion

**FIGURE 2:**
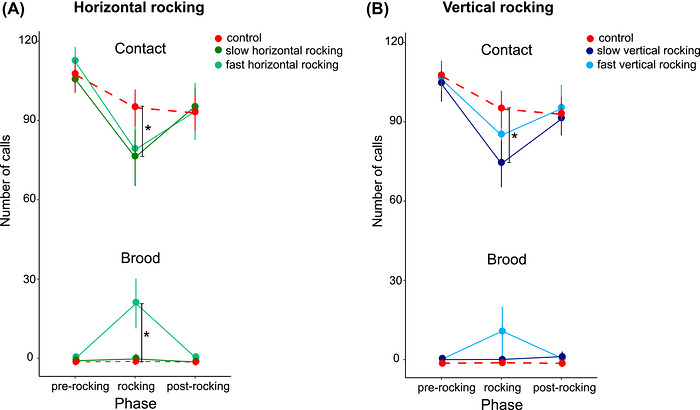
The effect of horizontal (panel A) and vertical (panel B) rocking conditions on the number of contact (on the top of each panel) and pleasure (on the bottom of each panel) calls emitted by chicks during the three experimental phases. The control group was the same for each rocking condition. Error bars represent ± 1 standard error. *indicates significant differences (*p* < 0.05).

Cohen's *κ* analysis revealed that inter‐rate agreement on call classification was very high, *κ* = 0.954. The ANOVA performed on contact calls (Figure 2, top of panels A and B) revealed a significant main effect of the phase, *F*(1.73, 224.64 = 85.34, *p* < 0.001, $\eta_{p}^{2}$ = 0.40), and a significant phase × rocking condition interaction, *F*(6.91, 224.64 = 2.72, *p* = 0.010, $\eta_{p}^{2}$ = 0.08). In all conditions, the number of contact calls decreased from the pre‐rocking to the post‐rocking phase. However, during the rocking phase, chicks in the slow rocking conditions significantly decreased the number of contact calls compared to the control condition (for the horizontal movement: *t*(130) = −2.84, *p* = 0.040; for the vertical movement: *t*(130) = −3.07, *p* = 0.021). No other difference across phases or conditions was significant.

For what concerns the brood calls analysis (Figure 2, bottom of panels A and B), the ANOVA revealed a significant main effect of the phase, *F*(1.05, 135.89 = 20.99, *p* < 0.001, $\eta_{p}^{2}$ = 0.14), the rocking condition, *F*(4, 130 = 87.86, *p* < 0.001, $\eta_{p}^{2}$ = 0.19), and a significant phase × rocking condition interaction, *F*(4.18, 135.89 = 8.80, *p* < 0.001, $\eta_{p}^{2}$ = 0.21). Specifically, while the number of brood calls was similar in all rocking conditions during the pre‐rocking and post‐rocking phase, during rocking, chicks in the fast horizontal condition produced significantly more brood calls than the control condition, *t*(130) = 4.29, *p <* 0.001, the slow horizontal rocking condition, *t*(130) = 4.86, *p <* 0.001, and the slow vertical rocking condition, *t*(130) = 4.41, *p =* 0.002, but not compared to the fast vertical rocking condition, *t*(130) = 2.31, *p* = 0.145.

Results show that contact and brood calls were differently affected by rocking. Contact calls decreased across experimental phases, and the decrease was significantly larger under slow rocking compared to the control condition. Brood calls increased during the fast horizontal rocking condition but not during fast vertical rocking or slow rocking (on both axes) compared to the control condition.

Yet, in naturalistic contexts such as maternal brooding or transport, animals are rarely exposed to such movements in isolation; rather, they typically experience a compound form of stimulation that combines vertical and horizontal displacements (e.g., carrying or walking). Moreover, nothing is known about the possible shift from soothing to pleasurable experience.

In the next experiment, we, therefore, examined how chicks respond to simultaneous stimulation along the vertical and horizontal axes during passive rhythmic and carrying‐like movement.

## 3 Experiment 2

### 3.1 Materials and Methods

#### 3.1.1 Animals

We observed 75 two‐day‐old domestic chickens (*Gallus gallus domesticus*, Ross 308 broiler strain, Aviagen); 37 chicks were female, and 38 chicks were male. Hatching and rearing conditions were identical to those of Experiment 1.

#### 3.1.2 Procedure

The procedure was like Experiment 1, with the following exception. The robotic arm moved simultaneously along the horizontal and vertical axes, following a sinusoidal‐like movement. In the fast‐carrying condition, the arm oscillated at a frequency of 2 Hz with a sinusoidal‐like amplitude of 2 cm, for a total displacement of 100 cm. In the slow‐carrying condition, the oscillation frequency was 0.2 Hz with a sinusoidal‐like amplitude of 20 cm for the same displacement. In these motions, there is no acceleration along the horizontal axis since the system moves at a constant speed, while along the vertical axis, the accelerations are the same as those set in the first experiment, namely, 316 cm/s^2^ for the fast rocking and 32 cm/s^2^ for the slow rocking. To comply with these values and ensure a horizontal stroke of 100 cm, six complete cycles of sinusoidal motion were adopted for slow motion and 60 for fast motion.

#### 3.1.3 Data Analysis

Data analysis was like in Experiment 1, with the exception that now there was a between‐subjects carrying condition including two levels (fast and slow), in addition to the within‐subjects phase condition (pre‐carrying still, carrying, post‐carrying still).

### 3.2 Results and Discussion

**FIGURE 3:**
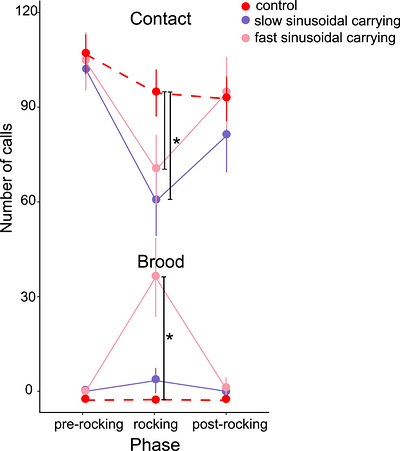
The effect of fast and slow sinusoidal movements on the number of contact (on the top) and pleasure (on the bottom) calls emitted by chicks during the three experimental phases. Error bars represent ±1 standard error. * indicates significant differences (*p* < 0.050).

The ANOVA performed on contact calls (Figure 3, top) revealed a significant main effect of the phase, *F*(1.97, 136.11 = 54.47, *p* < 0.001, $\eta_{p}^{2}$ = 0.44), carrying condition, *F*(2, 69 = 5.29, *p* = 0.007, $\eta_{p}^{2}$ = 0.13), and a significant phase × carrying condition interaction, *F*(3.95, 136.11 = 6.68, *p* < 0.001, $\eta_{p}^{2}$ = 0.16). While the number of contact calls decreased from the pre‐carrying to the post‐carrying phase in all conditions, both the fast and the slow carrying significantly reduced chicks’ contact calls compared to the control condition (for the fast‐carrying: *t*(69) = −3.34, *p* = 0.003; for the slow‐carrying: *t*(69) = −4.86, *p <* 0.001). No other difference across phases or conditions was significant.

For what concerns the brood calls analysis (Figure 3, bottom), the ANOVA revealed a significant main effect of the phase, *F*(1.13, 77.90 = 40.23, *p* < 0.001, $\eta_{p}^{2}$ = 0.37), the carrying condition, *F*(2, 69 = 25.65, *p* < 0.001, $\eta_{p}^{2}$ = 0.42), and a significant phase × carrying condition interaction, *F*(2.26, 77.90 = 28.92, *p* < 0.001, $\eta_{p}^{2}$ = 0.45). Specifically, the fast‐carrying condition increased the number of brood calls compared to the slow‐carrying condition, *t*(69) = 6.35, *p <* 0.001 and the control condition, *t*(69) = 7.15, *p <* 0.001.

Experiment 2 extended the findings of Experiment 1 by examining chicks’ responses to carrying‐like movement that simultaneously engaged the vertical and horizontal axes. The results revealed that both slow and fast sinusoidal‐like movements reduced contact calls relative to control, while fast sinusoidal‐like movement selectively enhanced brood calls.

## 4 General Discussion

Our findings show that rhythmic passive movement modulates chicks’ vocalizations and can induce positive affective states in a bird species that lacks any species‐specific experience of maternal rocking or carrying.

Slow rocking, whether horizontal or vertical, reduced the number of contact calls, producing a tangible soothing effect. Similarly, slow carrying, which integrates motion along both axes, yielded a comparable calming outcome, suggesting that the integration of multiple movement directions does not necessarily enhance the effect, and that the tempo of stimulation may be more critical than its complexity. It may be that rhythmicity itself, rather than the specific trajectory of movement, is the key factor, possibly because predictable rhythmic input reduces uncertainty and facilitates anticipatory regulation of bodily states, in line with predictive coding accounts of sensory processing [33]. From this perspective, the soothing impact of rhythmic passive motion could reflect the alignment between external periodicity and the organism's internal models of expected sensory flow, as also suggested by recent work on rhythm, prediction, and pleasurable states [34].

Directly related to predictive processing and theories of music preference, an innovative finding of our study is the increase in brood calls during fast horizontal rocking and fast carrying that indicates a transition from mere soothing effects toward a positively valenced and pleasurable state. This result is extremely relevant because it also helps clarify the concurrent attenuation of other vocalizations during movement: specifically, the increase in brood calls during motion indicates that the reduction of contact calls cannot be fully attributed to surprise, novelty, or mechanical interference with vocal production during motion, as chicks clearly retained, and selectively expressed, the capacity to vocalize, by changing calls in their valence. Indeed, chicks are known to vocalize robustly during active behaviors, including running and wheel locomotion. Moreover, in our experiments, they did not emit trills, that is, specific vocalizations typically associated with surprise [23]. While we cannot entirely exclude a transient contribution of novelty, particularly at movement onset, the differential vocal patterns observed here are more consistent with a modulation of affective state rather than purely attentional or mechanical constraints.

We acknowledge that, in the absence of an irregular or unpredictable movement control condition, it is not possible to definitively dissociate the contribution of temporal predictability from movement *per se*, nor to exclusively attribute the observed effects to rhythmic motion; we, therefore, interpret temporal predictability as a plausible contributing mechanism rather than an exclusive explanation. Consistent with this view, previous work has shown that chicks working to rejoin an artificial maternal stimulus respond more vigorously to fast, pulsating sequences of clucks than to slower or continuous ones, suggesting that the temporal organization of sensory input can modulate arousal and behavioral engagement [35].

At the same time, since previous studies have claimed that maternal carrying would induce a strong calming response in both humans and mice infants, and because carrying indubitably involves a rhythmic locomotor activity in these species, our results do show instead that such regulator effect does not depend on the physical contact characterizing the mother–infant interaction, as a comparable modulation of vocal behavior was observed in chicks, a species that does not naturally experience maternal transport.

More broadly, across species, and including humans, passive and active forms of repetitive or accelerated movement, such as rocking, swinging, or locomotor play, are often associated with behavioral indicators of positive engagement and reward. These observations align with the comparative perspective emphasizing the role of structured movement in shaping affective experience and social interaction, as discussed in behavioral and developmental frameworks [16, 36].

Taken together, our findings support the view that passive movement can modulate affective state in young animals, while highlighting the need for future studies specifically designed to disentangle the relative contributions of movement dynamics, predictability, and sensory integration.

It is also worth noting that we did not prolong the stimulation until the onset of sleep, if it occurred, as reported in other species. Future studies should examine whether extended passive movement promotes sleep induction [11, 22] and investigate how the temporal dynamics of positive vocalizations are modulated by prolonged exposure. Addressing these questions will not only clarify the boundaries between soothing and pleasurable states but also inform applications of rhythmic stimulation in the context of animal welfare.

## 5 Conclusion

Our findings expand the functional scope of rhythmic stimulation, positioning it as a regulator of affect with both calming and pleasurable dimensions across vertebrate lineages. The evidence that passive rhythmic motion can induce a comparable soothing response in chicks, despite the absence of maternal carrying in their natural history, suggests that sensitivity to rhythmic locomotor cues may be a conserved feature of vertebrate physiology. Rather than reflecting a byproduct of specific parental strategies, this response likely represents a more general mechanism for modulating arousal through movement, plausibly linked to predator avoidance, social cohesion, biological motion, or the rewarding properties of rhythmic stimulation. From this perspective, the soothing effects of carrying and rocking in mammals may have co‐opted an ancient and widespread sensitivity to rhythm and motion, which later acquired specialized functions in parent−offspring interactions.

## Author Contributions

C.C. conceived the study. C.C., A.D., L.S., and P.G. designed the experiments. A.D. collected and analyzed the data. C.C. and A.D. drafted the manuscript. All authors approved the final version of the manuscript.

## Conflicts of Interest

We have no conflicts of interest to declare.

## Ethics Approval Statement

This study complies with the current European and Italian laws for the ethical treatment of animals in research and has been approved by the Organismo Preposto al Benessere Animale of the University of Trieste and licensed by the Italian Health Ministry (permit 229/2017‐PR).

## Supporting information




**Supplementary Material**: nyas70321‐sup‐0001‐SuppMat.xlsx

## Data Availability

All raw data generated in this study are included in this published article as Supplementary Materials file (Raw_data.xlsx).
